# Single-cell RNA-Seq Elucidates the Crosstalk Between Cancer Stem Cells and the Tumor Microenvironment in Hepatocellular Carcinoma

**DOI:** 10.7150/jca.92185

**Published:** 2024-01-01

**Authors:** Sen Lin, Danfei Li, Yan Yang, Mengjiao Yu, Ruiqi Zhao, Jinghao Li, Lisheng Peng

**Affiliations:** 1 The Fourth Clinical Medical College, Guangzhou University of Chinese Medicine, Guangzhou, China.; 2 Department of Traditional Chinese Medicine, The Sixth Affiliated Hospital, South China University of Technology, Foshan, China.; 3 Department of Hepatology, Shenzhen Traditional Chinese Medicine Hospital, Shenzhen, China.

**Keywords:** single-cell RNA sequencing, cancer stem cells, tumor microenvironment, cell communication, crosstalk

## Abstract

**Background:** The challenge of systemic treatment for hepatocellular carcinoma (HCC) stems from the development of drug resistance, primarily driven by the interplay between cancer stem cells (CSCs) and the tumor microenvironment (TME). However, there is a notable dearth of comprehensive research investigating the crosstalk between CSCs and stromal cells or immune cells within the TME of HCC.

**Methods:** We procured single-cell RNA sequencing (scRNA-Seq) data from 16 patients diagnosed with HCC. Employing meticulous data quality control and cell annotation procedures, we delineated distinct CSCs subtypes and performed multi-omics analyses encompassing metabolic activity, cell communication, and cell trajectory. These analyses shed light on the potential molecular mechanisms governing the interaction between CSCs and the TME, while also identifying CSCs' developmental genes. By combining these developmental genes, we employed machine learning algorithms and RT-qPCR to construct and validate a prognostic risk model for HCC.

**Results:** We successfully identified CSCs subtypes residing within malignant cells. Through meticulous enrichment analysis and assessment of metabolic activity, we discovered anomalous metabolic patterns within the CSCs microenvironment, including hypoxia and glucose deprivation. Moreover, CSCs exhibited aberrant activity in signaling pathways associated with lipid metabolism. Furthermore, our investigations into cell communication unveiled that CSCs possess the capacity to modulate stromal cells and immune cells through the secretion of MIF or MDK, consequently exerting regulatory control over the TME. Finally, through cell trajectory analysis, we found developmental genes of CSCs. Leveraging these genes, we successfully developed and validated a prognostic risk model (APCS, ADH4, FTH1, and HSPB1) with machine learning and RT-qPCR.

**Conclusions:** By means of single-cell multi-omics analysis, this study offers valuable insights into the potential molecular mechanisms governing the interaction between CSCs and the TME, elucidating the pivotal role CSCs play within the TME. Additionally, we have successfully established a comprehensive clinical prognostic model through bulk RNA-Seq data.

## Background

Liver cancer, characterized by its high malignancy, rapid progression, and significant mortality and disability rates, poses a serious threat to human health. The latest global cancer report estimated approximately 900,000 new cases of liver cancer in 2020, resulting in approximately 830,000 deaths^1^.Liver cancer ranks second, following lung cancer, in terms of mortality rate and number of deaths, resulting in an extremely poor prognosis. Hepatocellular carcinoma (HCC) is the predominant pathological type of liver cancer, accounting for approximately 85% of cases. The overall 5-year survival rate for HCC is below 20%, further decreasing for patients with advanced disease^2^, ^3^.

The dilemma in the systemic treatment of HCC primarily stems from the development of drug resistance, which is rooted in the heterogeneity of tumor cells^4^-^6^. Traditional bulk sequencing techniques are limited in their ability to comprehensively study tumor heterogeneity, posing significant challenges in drug development and the detection of diagnostic markers. Single-cell RNA sequencing (scRNA-Seq), spatial transcriptomics, and single-cell proteomics enable the analysis of heterogeneity across distinct cell populations, uncovering the interplay between tumor cells and the tumor microenvironment (TME), comprising lymphatic or vascular endothelial cells, immune cells, fibroblasts, various signaling molecules, and the extracellular matrix.^7^-^10^. Cancer stem cells (CSCs) constitute a minor subset of undifferentiated cells in tumor tissue, with robust self-renewal potential and tumorigenic capabilities, crucially contributing to tumor heterogeneity^11^. Continuous clonal proliferation in CSCs confers them with robust immune evasion abilities and drug resistance, contributing to treatment failure, tumor recurrence, and metastasis in systemic therapies^12^, ^13^. The influence of CSCs on tumor heterogeneity stems from their robust self-renewal capabilities and their interactions with the TME^14^, ^15^, including crosstalk with stromal and immune cells. These interactions activate signaling pathways like PI3K/AKT/mTOR and TGFβ, promoting tumor progression. Previous studies have shown the feasibility and benefits of utilizing scRNA-Seq to investigate tumor heterogeneity and analyze the landscape of single-cell transcriptomes^16^. Nevertheless, further comprehensive and in-depth research on CSCs is necessary, despite the maturity of scRNA-Seq and the development of algorithms for analyzing multi-dimensional data. Hence, this study seeks to employ scRNA-Seq to analyze cell communication, cellular developmental trajectories, and metabolic activities, aiming to identify genes associated with CSCs development. Additionally, through the integration of bulk RNA-Seq data, we have successfully established a comprehensive clinical prognostic model.

## Methods and Materials

### Quality Control and Normalization of scRNA-Seq Data

The single-cell RNA sequencing (scRNA-Seq) data generated and analysed during the current study are available in the Mendeley Data (https://data.mendeley.com/datasets/skrx2fz79n/1)^17^ and the Gene Expression Omnibus (GEO) database (https://www.ncbi.nlm.nih.gov/geo/query/acc.cgi?acc=GSE149614)^18^, with sequencing performed on the Illumina NovaSeq 6000 platform. Upon importing the data into R (v4.2.2) using the Seurat package (v4.3.0)^19^, data merging was conducted, and the sequencing depth was ascertained. Quality control procedures were then implemented on the merged data, taking into account the RNA molecule count per cell (nFeature), total gene expression per cell (nCount), and the percentage of mitochondrial genes (percent.mt) present within each cell. The following filtering criteria were employed: 1000 < nCount < 10000, 250 < nFeature < 4000, percent.mt < 15%. Subsequently, the data underwent normalization using SC-Transform, and batch effects were corrected by integrating samples with the harmony package (v0.1.1)^20^ in R.

### Dimensionality Reduction and Clustering

In this study, we employed dimensionality reduction techniques and clustering methods to analyze the data. Initially, we performed principal component analysis (PCA) on the quality-controlled dataset. The scree plot was used to determine the appropriate number of dimensions for PCA, which were subsequently used in the analysis. To establish the relationships between cells, we calculated the k-nearest neighbors (KNN) and constructed a shared nearest neighbor graph. The Louvain algorithm was then applied to optimize the modularity function and identify distinct cell clusters. For further exploration and visualization, we utilized the clustree package (v0.5.0)^21^ in R to determine an optimal resolution range (0.5-1.2) for defining specific cell clusters. Additionally, to enhance dimensionality reduction, we employed t-distributed stochastic neighbor embedding (tSNE) on the PCA-transformed data and generated visual representations of the cell populations.

### Cell Annotation

Cell types were annotated based on the clustering results, followed by the annotation of cell subtypes. This study employed a combined approach, using automated annotation with the SingleR package (v1.10.0)^22^ in R, along with manual annotation based on literature, to annotate both cell types and subtypes. Prior to cell type annotation, differential expression genes (DEGs) analysis was conducted for each cell type using the FindMarkers function in the Seurat package. DEGs were selected based on the criteria of Log2 fold change (Log2Fc) > 0.5 and Benjamini-Hochberg-adjusted *p*-values < 0.05. Subsequently, automated annotation based on DEGs was performed using SingleR, and the results were manually curated for refinement.

Next, Seurat objects representing different cell types were extracted based on the annotated cell types for further dimensionality reduction using PCA and tSNE. This process led to the clustering of cell subtypes. The aforementioned steps for cell type annotation were repeated for cell subtype annotation, with a greater emphasis on manual annotation. Finally, tSNE visualization was conducted on the annotated cell data, presenting the proportions of different cell types and cell subtypes, as well as the expression levels of marker genes.

### Enrichment Analysis and Metabolism Analysis

Differential gene expression analysis was performed on each cell cluster using the FindMarkers function in the Seurat package. The Wilcoxon rank-sum test was applied with criteria of Log2 fold change (Log2Fc) > 0.5 and Benjamini-Hochberg-adjusted *p*-values < 0.05 to select DEGs for each cluster. A subset of DEGs was then chosen for visualization using heatmaps. Similarly, DEGs were selected for each cell type and visualized through heatmaps.

Subsequently, Gene Set Enrichment Analysis (GSEA) and Gene Set Variation Analysis (GSVA) were conducted on each cell type using the fgsea package (v1.22.0)^23^ and GSVA package (v1.60.0)^24^ in R. Gene sets from the Gene Ontology (GO) database were employed to assess biological functions, while gene sets from the Kyoto Encyclopedia of Genes and Genomes (KEGG) and Hallmark databases were used for signaling pathway analysis. GSVA analysis was performed specifically on the six major cell clusters, unveiling differential biological functions and signaling pathways. Furthermore, differential gene expression analysis was carried out on the Malignant Hepatocytes and CSCs subtypes within the liver cell population. DEGs were utilized for focused GSEA enrichment analysis (GO and KEGG) as well as GSVA enrichment analysis (Hallmark). Finally, metabolism analysis of the liver cell subtypes was conducted using the scMetabolism package (v2.1.0)^25^ in R to evaluate the activity of metabolic pathways in both malignant and non-malignant cells. Gene sets for metabolic pathways were sourced from the KEGG and REACTOME databases. Metabolic activity scores were calculated for each cell using the VISION algorithm^26^, and selected key metabolic pathways were visualized using a bubble plot.

### Cell Communication Analysis

We performed an analysis of cell-cell communication using the CellChat package (v1.6.1)^27^ in R. Our aim was to determine the communication status of ligand-receptor pairs between cells. To achieve this, we utilized the CellChat algorithm to assess the contributions of ligand-receptor pairs, both in terms of their output and input, to various signaling pathways. By doing so, we were able to estimate the probability or strength of communication at the signaling pathway level among different cell types. To visualize the cell-cell communication networks, we relied on the probabilities or strengths of communication. Furthermore, we employed unsupervised learning through non-negative matrix factorization (NMF)^28^ to identify distinctive communication patterns within cells. This approach facilitated the recognition of coordinated communication patterns, including both outgoing and incoming interactions, across multiple cell types and signaling pathway levels.

### Copy Number Variation Analysis

In the preceding sections, we performed subtype annotation of hepatocytes through a combination of automated and manual methods. Based on this annotation, we segregated the cells into two clusters: malignant and non-malignant. To validate the accuracy of our hepatocytes subtype annotation, we employed the inferCNV package (v1.16.0) in the R programming language to conduct an analysis of copy number variation (CNV). For additional details, please refer to the following link: https://github.com/broadinstitute/inferCNV. By utilizing a Hidden Markov Model (HMM) for prediction and “ward.D2” hierarchical clustering, we generated visual representations, using the pheatmap package (v1.0.12) in R, to illustrate the CNV profiles of both malignant and non-malignant cells. These visualizations highlighted the presence of deletions, amplifications, or absence of variations, enabling a clear differentiation between malignant and non-malignant cells. Furthermore, the CNV profiles provided valuable insights into the heterogeneity among malignant cells. Subsequently, we normalized the expression levels of each cell and calculated the sum of squared normalized values, yielding the CNV scores.

### Cell Trajectory Analysis

In the preceding sections, we conducted a re-clustering of hepatocytes following their subtype annotation, which enabled the identification of CSCs and HCC cells. To predict the cell's developmental trajectory from CSCs to HCC and identify genes associated with evolution and development, we performed pseudotime analysis using Monocle3. We employed the Monocle3 package (v1.3.1)^29^-^31^ in R, utilizing the SimplePPT algorithm for trajectory learning and an iterative algorithm for semi-supervised pseudotime analysis. This approach allowed us to construct a developmental trajectory plot of cells. Furthermore, we validated the cell's developmental trajectory by conducting Monocle2^29^, ^31^ pseudotime analysis with the DDRTree algorithm. For genes related to development (Moran's I > 0.5), we conducted enrichment analysis using fGSEA, following the same detailed analysis process described earlier. Lastly, we calculated the Moran's I index of genes using a spatial differential gene algorithm. The Moran's I index ranges from -1 to 1, where 1 indicates a strong positive correlation, and Moran's I less than or equal to 0 indicates no correlation. Based on the Moran's I index, we selected genes highly correlated with development (Moran's I > 0.8) and visualized their expression dynamics along the pseudotime trajectory, and their expression patterns on the UMAP dimensionality reduction plot.

### Weighted Gene Co-expression Network Analysis

We conducted a weighted gene co-expression network analysis (WGCNA) using the WGCNA package (v1.72-1)^32^, ^33^ in R to identify gene expression modules and explore their correlation with phenotypes. Initially, we applied a sample clustering tree algorithm to remove outlier samples, ensuring the stability of the co-expression network construction. Our objective was to establish a scale-free network by adjusting the soft threshold (power) from 1 to 30, guided by the scale-free topology fit index (>0.85) and average connectivity, employing the pickSoftThreshold function. Next, we transformed the adjacency matrix into a topological overlap matrix (TOM) to reduce spurious correlations and noise. 1-TOM was calculated as a significant biological indicator of interconnectivity within the co-expression network and as a distance metric for gene clustering. The dynamic tree cut algorithm was then utilized to identify gene modules, with each module containing a minimum of 50 genes. Moreover, we computed module eigengenes (MEs) that represent the characteristics of each module. These modules were hierarchically clustered, and those exhibiting similar patterns, identified by a cut height of 0.25, were merged and visually distinguished by different colors. Heatmaps were generated to visualize the expression profiles of MEs and to calculate module membership (MM, correlation between MEs and the expression profiles of all genes) and gene significance (GS, correlation between MEs and phenotypes). Furthermore, correlation heatmaps were created to illustrate the relationship between MEs and the expression profiles of all genes, as well as the correlation between modules and phenotypes. We identified modules associated with cell subtypes and generated heatmaps displaying the expression profiles of module-specific genes corresponding to each subtype.

### Construction and Validation of Clinical Prognostic Model

In the preceding sections, we identified cell trajectory-related genes (Moran's I > 0.5) using Monocle3 pseudotime analysis and cell subtype-related genes (MEturquoise module and MEblue module) through WGCNA analysis. By combining these two gene sets, we identified the intersection genes that represent genes associated with the development of CSCs. The bulk RNA-Seq data mentioned in the article were obtained from TCGA-LIHC (https://portal.gdc.cancer.gov/repository) and GSE76427 (https://www.ncbi.nlm.nih.gov/geo/query/acc.cgi?acc=GSE76427)^34^ for the subsequent construction and validation of the prognostic model.

Initially, the samples were randomly divided into training and validation cohorts. We conducted univariate Cox regression analysis on the aforementioned intersection genes to select genes associated with prognosis. Subsequently, we employed the Least Absolute Shrinkage and Selection Operator (LASSO) regression analysis with 10-fold cross-validation and multiple-factor Cox regression analysis to further refine the selection of prognostic-related genes and construct a risk scoring model. This model establishes the relationship between gene expression levels and prognosis using various coefficients and is represented by the following formula:



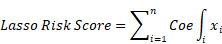



In the formula, 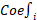 represents the correlation coefficient, and 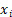 represents the normalized expression levels of genes associated with prognosis. Subsequently, we performed a validation of the risk scoring model to assess its accuracy. Initially, we conducted an analysis of survival status and survival time differences between high-risk and low-risk groups in the training cohort. The results were presented using scatter plots and Kaplan-Meier survival curves, which were then validated in the independent validation cohort. Next, we assessed the predictive capability of the risk scoring model for prognosis by analyzing the Area under the Curve (AUC) of Receiver Operating Characteristic (ROC) curves for 1-year, 3-year, and 5-year overall survival (OS) in both the training and validation cohorts. Lastly, by integrating clinical data with the risk scoring model, we developed Nomograms and evaluated the concordance between the predicted and actual values of 1-year, 3-year, and 5-year OS using Calibration curves.

### Real Time Quantitative Polymerase Chain Reaction (RT-qPCR) Validation

We conducted RT-qPCR to assess the expression levels of APCS, ADH4, FTH1, and HSPB1 in HepG2 cells and HepG2-CSCs. The HepG2-CSCs were enriched from the HepG2 cell line using serum-free tumor stem cell culture medium (the formulation refer to Supplementary Tables). RNA extraction was carried out from both HepG2 cells and HepG2-CSCs using the RNA/DNA Isolation Kit (Beyotime, China). Following cell lysis with Trizol, RNA was separated and extracted using a washing solution. Subsequently, cDNA was synthesized through a reverse transcription system (details provided in Supplementary Tables). The cDNA concentration was adjusted using RNase-free water, and the RT-qPCR analysis was performed utilizing the BeyoFast™ Probe One-Step RT-qPCR Kit (Beyotime, China). Further information regarding the RT-qPCR reaction system and program can be found in Supplementary Tables. The relative mRNA expression levels were computed using the 2^-(∆∆Ct) method.

## Results

### Quality Control and Normalization of scRNA-Seq data

The scRNA-Seq data were collected from two independent studies. Upon data integration and application of the aforementioned criteria, tumor tissue samples were selected, resulting in a total of 54,674 cells derived from 16 tumor tissue samples, the detailed clinic parameters of enrolled patients can be found in Supplementary Tables. The characteristics of the scRNA-Seq data before and after filtering are presented in Figure S1A and S1B, respectively. Utilizing tSNE for dimensionality reduction clustering, evident batch effects were observed among different samples, as illustrated in Figure S1C. To address this issue, the scRNA-Seq data underwent normalization and sample integration using the Harmony method to correct for batch effects. Figure S1D represents the sample features following batch effect correction.

### Dimensionality Reduction, Clustering and Cell Annotation

Based on the scree plot results (Figure S2A), we determined that 30 principal components (PCs) were appropriate, and performed PCA on the quality-controlled data. The results of the clustering tree (Figure S2B) demonstrated that a resolution of 0.8 produced satisfactory clustering outcomes. Subsequently, we employed tSNE for dimensionality reduction clustering, resulting in the formation of 24 clusters (Figure S2C). Furthermore, we computed the cell cycle phase for each cell (Figure S2D) and integrated it with the normalized sample features (Figure S2E), revealing a minimal impact of the cell cycle on the dimensionality reduction clustering. Finally, differential expression gene analysis for the identified 24 clusters was conducted using the Wilcoxon rank-sum test, and the heatmap in Figure S2F displays the expression levels of the top 5 DEGs.

**Figure 1:**
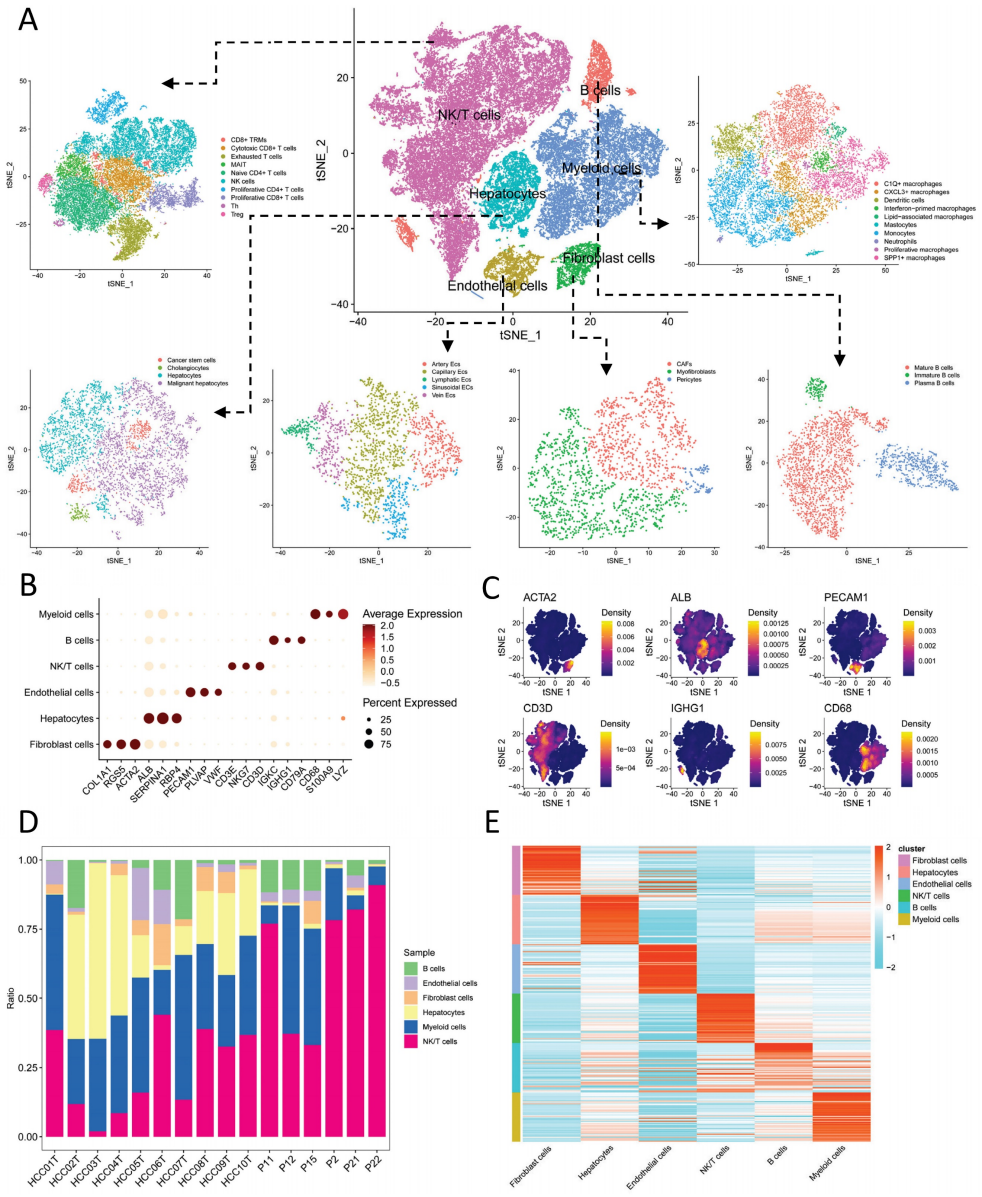
** Cell Annotation and Proportions.** A. t-SNE dimensionality reduction clustering plot displaying cell annotation results, identifying six main cell types, followed by subtypes annotation for each cell type; B. Dot plots presenting the average expression levels of markers used to identify the six main cell types; C. Density plot illustrating the expression profiles of markers after t-SNE dimensionality reduction clustering; D. Proportions of the six main cell types in each of the 16 samples; E. Heatmap depicting the expression levels of the top 50 differentially expressed genes in the six main cell types.

**Figure 2:**
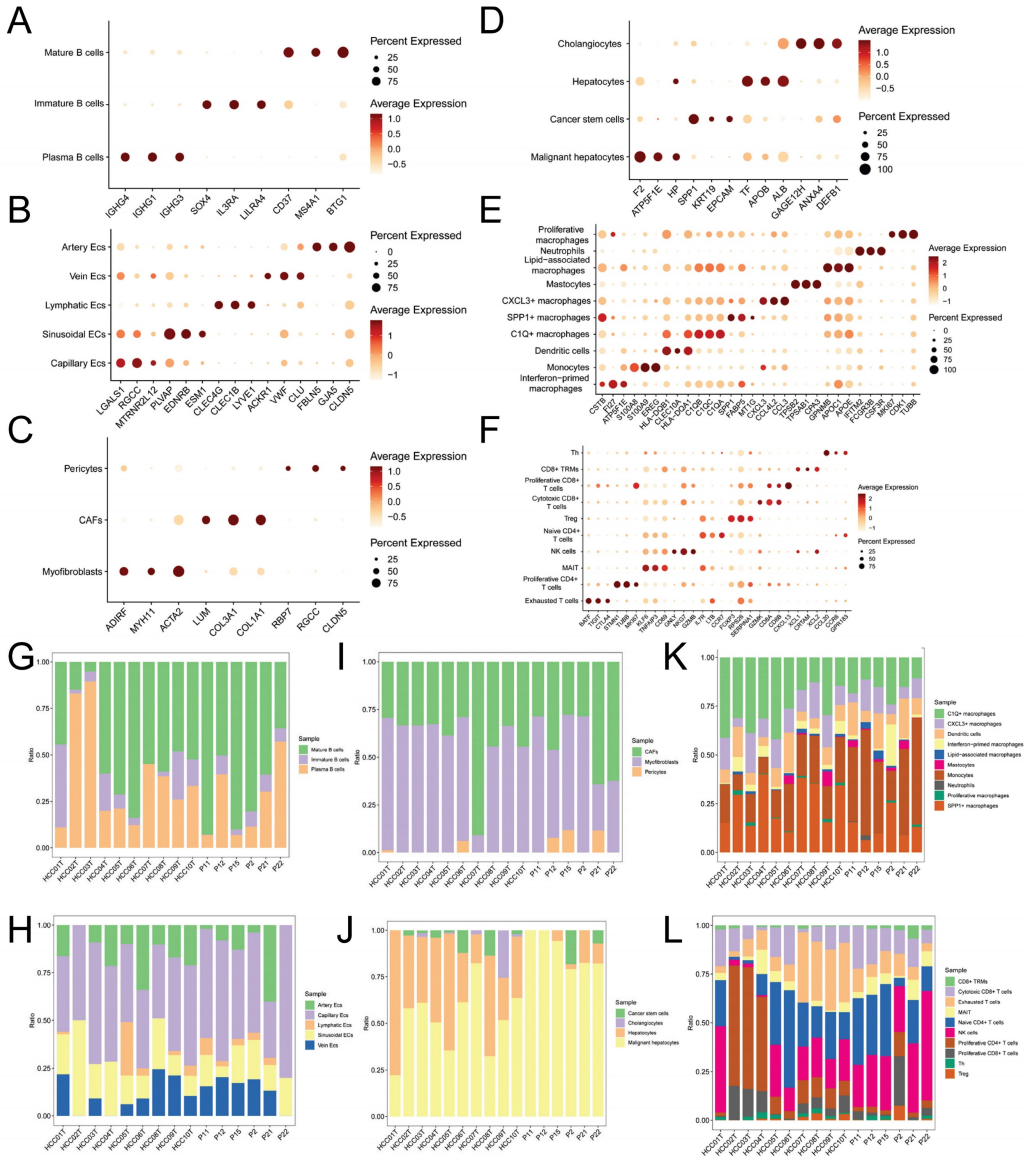
** Expression Levels of Subcluster Markers and Cell Counts.** A-F. Dot plots showing the average expression levels of subcluster markers for B cells, endothelial cells, fibroblasts, hepatocytes, myeloid cells, and NK/T cells, respectively; G-L. Bar plots illustrating the proportions of cell counts for each subcluster of B cells, endothelial cells, fibroblasts, hepatocytes, myeloid cells, and NK/T cells in each of the 16 samples.

We initially identified six main cell types based on markers (Figure 1A): B cells (IGKC, IGHG1, CD79A), endothelial cells (PECAM1, PLVAP, VWF), fibroblast cells (COL1A1, RGS5, ACTA2), hepatocytes (ALB, SERPINA1, RBP4), myeloid cells (CD68, S100A9, LYZ), and NK/T cells (CD3E, CD3D, NKG7). The expression levels of markers in cell types are shown in Figure 1B, while Figure 1C displays a tSNE density plot of marker expression. The proportion of the main cell types is illustrated in Figure 1D, with NK/T cells (NK/T) being the most abundant, followed by myeloid cells (Mye) and hepatocytes (Hep), while fibroblast cells (Fib) and endothelial cells (Endo) are less prevalent. We performed differential expression gene analysis, resulting in the identification of DEGs for the six major cell clusters. The heatmap in Figure 1E presents the expression levels of the top 50 DEGs. Subsequently, we conducted dimensionality reduction clustering for the six main cell types, and Figure S3A-F shows the tSNE plots of clustering results for each cell types, providing annotations for the subtypes of the main cell types (Figure 1A) along with their corresponding markers (Figure 2A-F). The proportions of cell subtypes can be observed in Figure 2G-L. Among NK/T cells, the proportion of exhausted T cells was the highest (Figure 2L), accompanied by high expression of immune checkpoint genes such as BATF, TIGIT, and CTLA4 (Figure 2F), suggesting immune evasion in the TME of HCC tissue. It is worth noting that due to the diverse functions of macrophages, an increasing number of macrophage subtypes have been discovered. Hence, in this study, we combined multiple studies to annotate macrophages into six subtypes (Figure 1A), including interferon-primed macrophages associated with interferon response, lipid-associated macrophages linked to lipid metabolism, and three macrophage subtypes characterized by the high expression of specific genes: CXCL3+ macrophages, SPP1+ macrophages, and C1Q+ macrophages. Additionally, proliferative macrophages were exhibiting high proliferative activity.

As there are numerous macrophage subtypes, the discrimination between them (Figure 2E) was not as distinct as the annotation results for B cells (Figure 2A), endothelial cells (Figure 2B), and fibroblast cells (Figure 2C). Regarding the annotation of hepatocyte types, the markers for normal hepatocytes were TF (Transferrin), ALB (Albumin), and APOB (Apolipoprotein B), which are important products of normal liver metabolisms. On the other hand, markers for malignant hepatocytes included F2 (Coagulation Factor II), ATP5F1E, and HP (Haptoglobin), all of which are indicative of abnormal metabolisms in malignant hepatocytes.

### Enrichment Analysis and Metabolic Analysis

**Figure 3:**
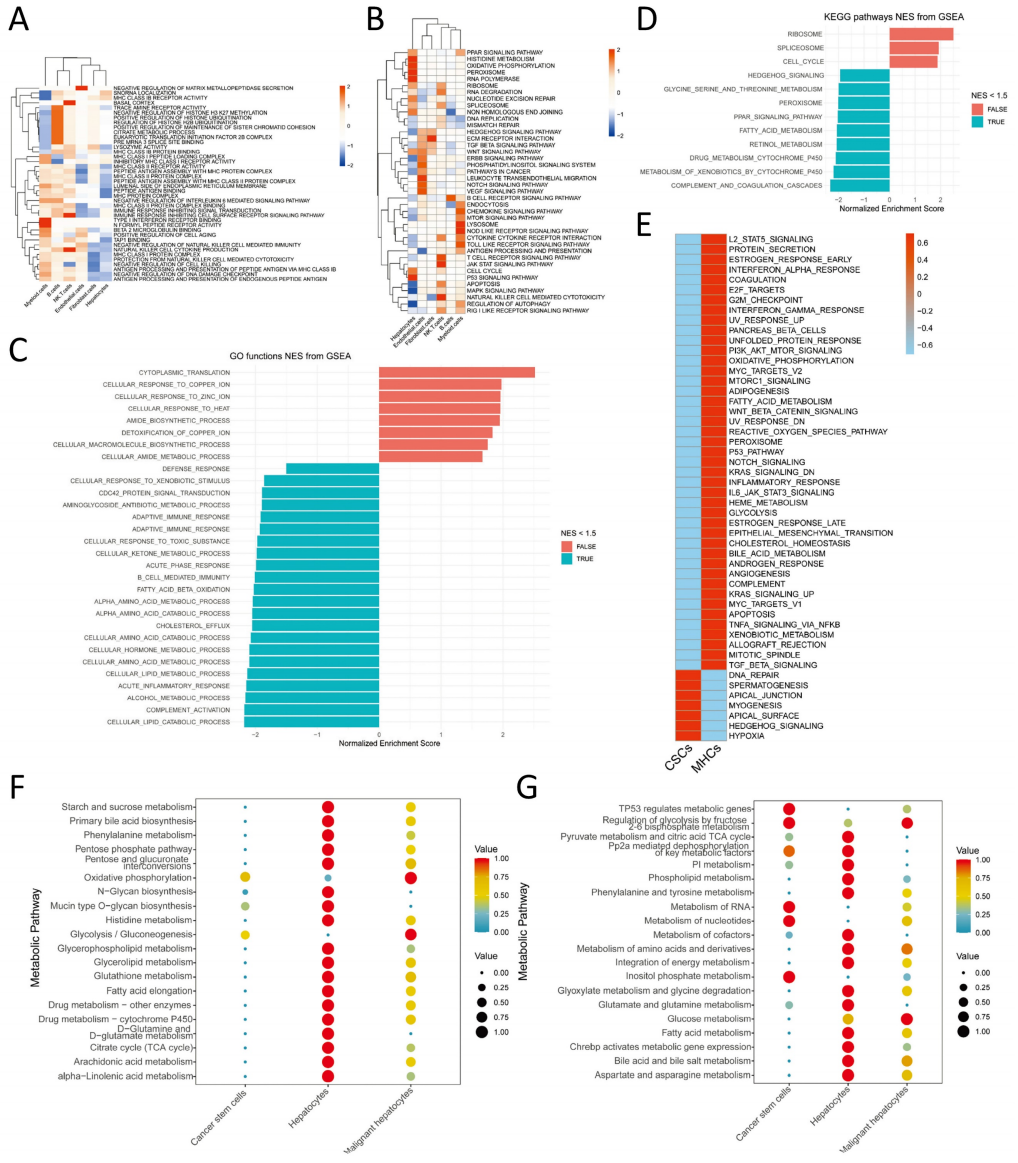
** Enrichment Analysis.** A. Gene set variation analysis (GSVA) for the enrichment analysis of biological functions in the main cell types; B. GSVA for the enrichment analysis of signaling pathways in the main cell types; C. Enrichment analysis of Gene Ontology (GO) biological functions in malignant cells using fGSEA; D. Enrichment analysis of Kyoto Encyclopedia of Genes and Genomes (KEGG) signaling pathways in malignant cells using fGSEA; E. Enrichment analysis of Hallmarker signaling pathways in malignant cells using GSVA; F-G. Enrichment analysis of hepatocytes subtypes for scMetabolism metabolic pathways based on KEGG and REACTOME, respectively.

In the preceding sections, we conducted differential gene expression analysis for the major cell clusters. Using GO, KEGG, and Hallmark gene sets, we performed enrichment analysis employing the fGSEA and GSVA algorithms. The results of the enrichment analysis for the main cell types are presented in Figure 3A-B. Our findings indicate significant enrichment of signaling pathways such as Wnt, TGF-β, and Hedgehog in Endothelial cells and Fibroblast cells, which are known to be involved in tumor development and progression. Moreover, Myeloid cells displayed significant enrichment in pattern recognition receptors (Toll-like receptor, RIG-I-like receptor, Nod-like receptor), cytokine-cytokine receptor interaction, and chemokine signaling pathways, suggesting their participation in both non-specific and specific immune responses. Additionally, Myeloid cells exhibited significant enrichment in the PPAR signaling pathway. Considering the annotation results of cell subtypes, we speculate that the PPAR signaling pathway plays a pivotal role in lipid metabolism within Lipid-associated macrophages. Hepatocyte types demonstrated notable enrichment in signaling pathways associated with nucleic acid, lipid, and protein metabolism, programmed cell death, and cell cycle. In the previous section, we classified the Hepatocyte cluster into four categories: Hepatocytes, Malignant hepatocytes, CSCs, and Cholangiocytes. We further conducted differential expression analysis for Malignant hepatocytes and CSCs, followed by enrichment analysis using DEGs. The fGSEA results (Figure 3C-D) revealed significant differences between the two groups in protein, lipid, and nucleic acid metabolism pathways, as well as pathways related to cell cycle. The enrichment results from GSVA (Figure 3E) provide a more comprehensive overview of the aforementioned findings. Notably, CSCs exhibited a significant association with Hedgehog and Hypoxia signaling pathways.

In light of the preceding analysis, our investigation focused on exploring metabolic disparities among distinct subtypes of Hepatocytes type. Following the exclusion of the Cholangiocytes subtype, we performed scMetabolism analysis using the KEGG and REACTOME gene sets. The findings (Figure 3F-G) revealed that normal hepatocytes manifested heightened activity across the majority of metabolic pathways to fulfill their physiological metabolic requirements. In contrast, Malignant hepatocytes exhibited elevated activity in the oxidative phosphorylation and glycolysis/gluconeogenesis pathways, while demonstrating moderate activity in other metabolic pathways. CSCs exhibited diminished overall metabolic activity but displayed increased engagement in nucleotide metabolism and glycolysis/gluconeogenesis, as well as involvement in inositol phosphate and phosphoinositide metabolism.

### Cell Communication

**Figure 4:**
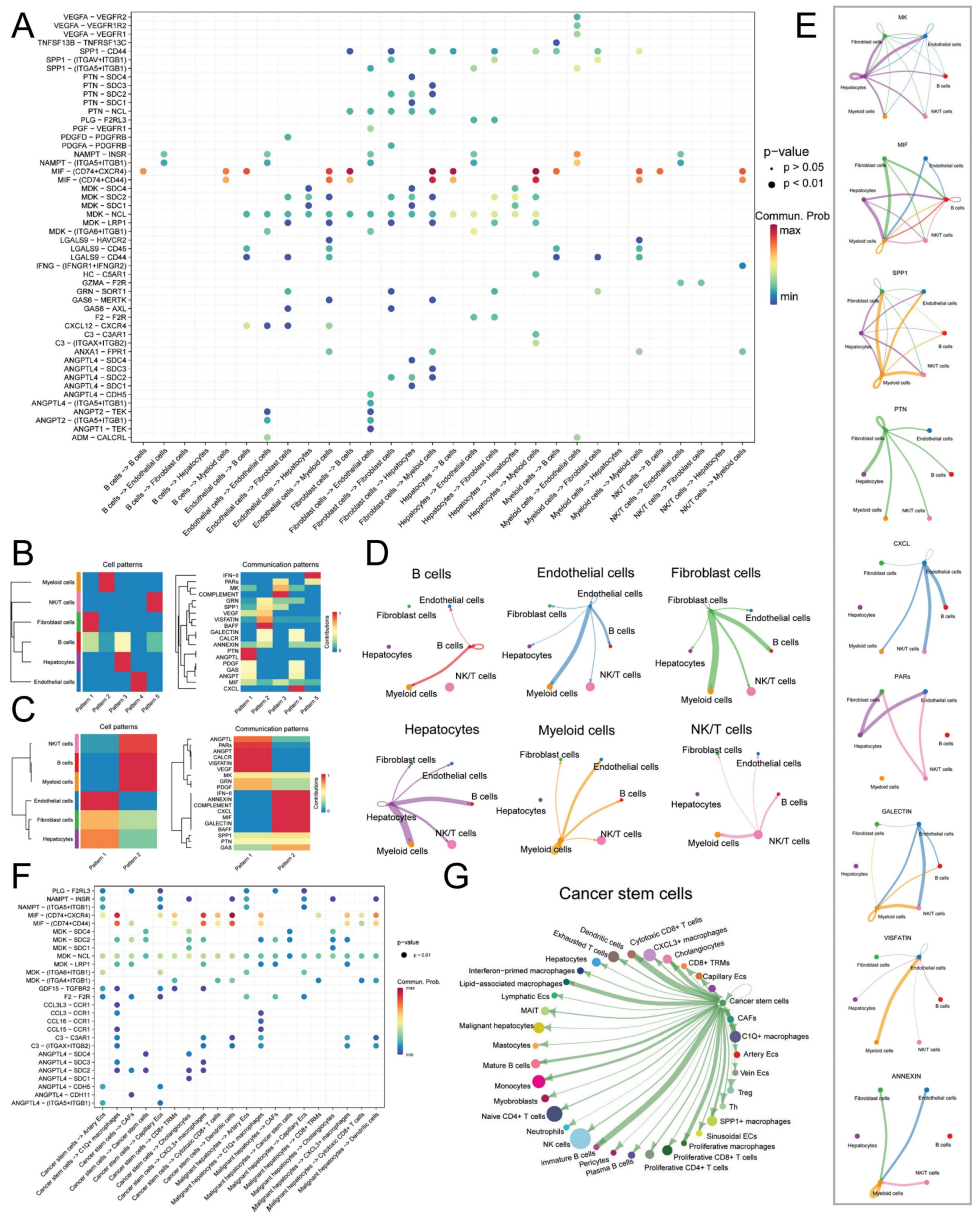
** Cell Communication Network of Main Cell Types.** A. Dot plot illustrating the ligand-receptor cell communication network between main cell types, with the color and size of the dots representing the likelihood and corresponding p-value of ligand-receptor-mediated cell communication; B. Non-negative matrix factorization (NMF) identifying incoming communication patterns of cells; C. NMF identifying outgoing communication patterns of cells; D. Cell communication network of the six main cell types; E. Ligand-mediated cell communication network, including MK, MIF, SPP1, PTN, CXCL, PARs, GALECTIN, VISFATIN, and ANNEXIN; F. Dot plot showing the ligand-receptor pairs of cell communication network between malignant hepatocytes and tumor stem cells; G. Outgoing communication network of CSCs.

Initially, we performed an analysis of ligand-receptor pair communication among the main cell types. The results, as depicted in Figure 4A, revealed significant ligand-receptor interactions among the main cell types. Endothelial cells, Fibroblast cells, and Hepatocytes, serving as the main signal-outgoing cells, displayed highly active regulatory networks to B cells, NK/T cells, and Myeloid cells. Several cell communication pathways were identified among the following pairs: Endo-Fib, Endo-Mye, Fib-Endo, Fib-Hep, Fib-Mye, and Hep-Mye. The prominent ligand-receptor pairs implicated in these pathways encompassed MIF-CD74/CXCR4, MIF-CD74/CD44, MDK-NCL, MDK-SDC2, and MDK-SDC4. Through the application of NMF clustering for dimensionality reduction, we discerned distinct patterns of ligand-receptor pair communication among the main cell types (Figure 4B-C). The findings suggested that Endothelial cells, Fibroblast cells, and Hepatocytes exhibited a shared pattern as signal-outgoing cells, characterized by primary ligands such as IFN-Ⅱ, ANNEXIN, MIF, and CXCL. Conversely, B cells, NK/T cells, and Myeloid cells displayed a distinct pattern and were regulated by PARs, VISFATIN, and MK. In terms of the input mode, a distinct clustering pattern was observed solely between B cells and Hepatocytes, whereas the remaining clusters received signals through diverse modes. Significantly, Macrophage Migration Inhibitory Factor (MIF) and Midkine (MDK), as ligands, exerted a substantial influence on the communication networks of multiple cell types, with specific ligand-mediated cell communication networks illustrated in Figure 4D-E.

Subsequently, we isolated Malignant hepatocytes and CSCs for further analysis of cell communication. The results (Figure 4F) revealed that Malignant hepatocytes and CSCs exhibited high activity in signal emission. Through different secreted factors, such as MIF and MDK, they participated in the regulation of various subtypes of Endothelial cells, Fibroblast cells, NK/T cells, and Myeloid cells, including C1Q+ macrophages (MIF-CD74/CXCR4, MIF-CD74/CD44, MDK-NCL, etc.), CXCL3+ macrophages (MIF-CD74/CXCR4, MIF-CD74/CD44, MDK-NCL, etc.), cancer-associated fibroblasts (CAFs) (F2-F2R, MDK-SDC2, MDK-NCL, etc.), Cytotoxic CD8+ cells (MIF-CD74/CXCR4, MIF-CD74/CD44, MDK-NCL, etc.), and Dendritic cells (MIF-CD74/CXCR4, MIF-CD74/CD44, etc.). CSCs exhibited higher activity than Malignant hepatocytes in signal emission, particularly in the regulation of C1Q+ macrophages and CXCL3+ macrophages. The cell communication network of CSCs is shown in Figure 4G.

### Copy Number Variation and Cell Trajectory Analysis

**Figure 5:**
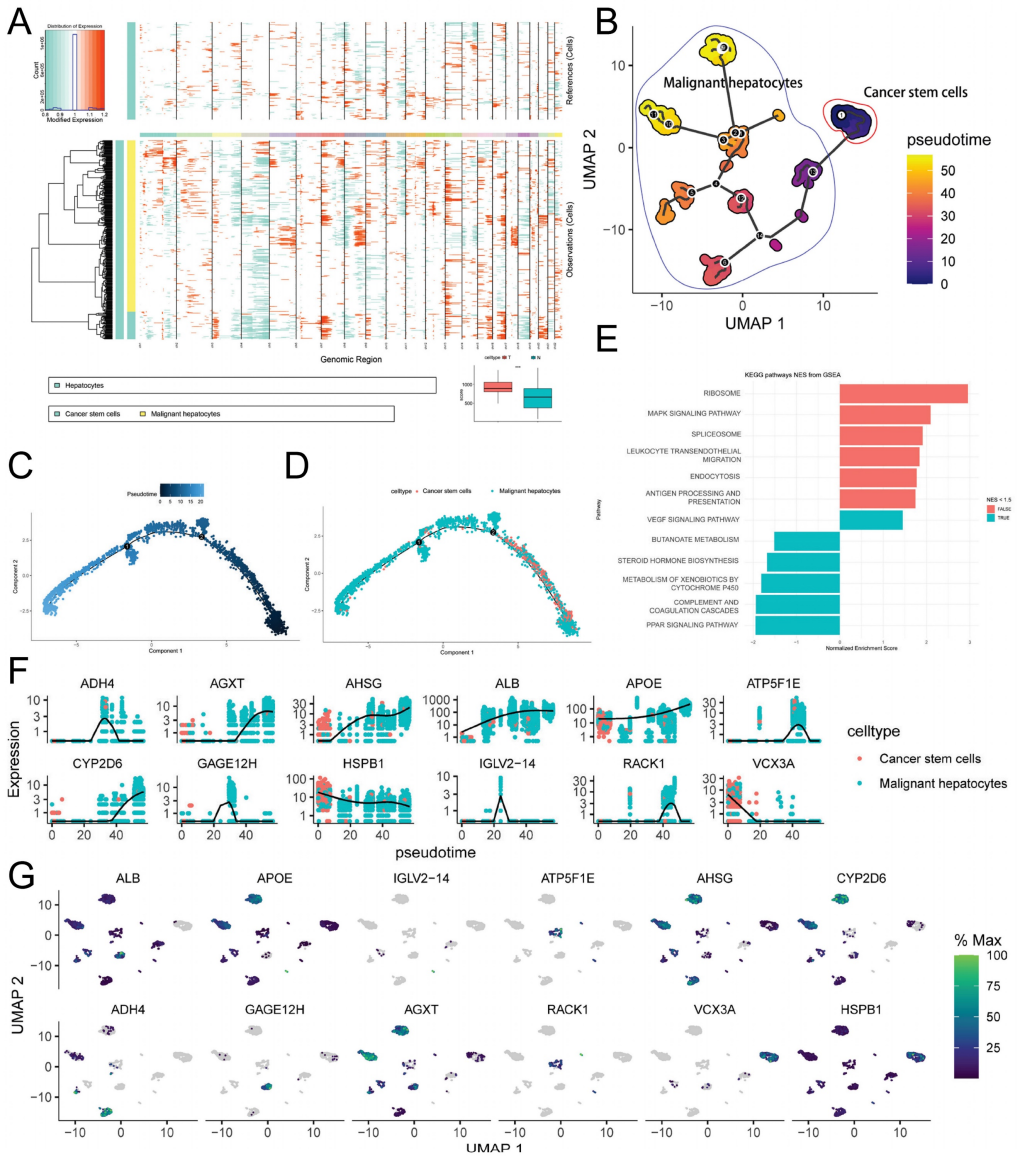
** Copy Number Variation and Cell Trajectory Analysis.** A. inferCNV heatmap displaying gene copy number variations (CNVs) in normal hepatocytes and malignant hepatocytes, with red indicating relative gene amplification and cyan indicating relative gene deletion. The bottom right corner shows the comparison of CNV scores between normal hepatocytes and malignant hepatocytes (*** p-value < 0.001); B. Monocle3 pseudotime analysis of malignant hepatocytes, presented as a UMAP dimensionality reduction plot; C-D. Monocle2 pseudotime analysis of malignant hepatocytes, showing the results of pseudotime analysis (C) and the distribution of hepatocytes subtypes (D) using DDRTree dimensionality reduction; E. Enrichment analysis of CSCs development-related genes (Moran index > 0.5) using fGSEA; F-G. Expression level changes of selected genes associated with CSCs development during Monocle3 pseudotime analysis (F), visualized using a UMAP dimensionality reduction plot (G).

Using the inferCNV package in R (v1.16.0), we conducted CNV analysis. The results (Figure 5A) revealed that CNV in normal hepatocytes was evenly distributed across chromosomes within the hepatocytes type. In contrast, malignant cells exhibited an uneven distribution of CNV across chromosomes, with significant variation among different cells. Notably, some malignant cells demonstrated lower CNV, indicating a high level of heterogeneity within the malignant cell population. However, the overall CNV score of malignant cells was significantly higher than that of normal cells (Figure 5A), thereby supporting the rational annotation of hepatocyte subtypes presented in previous sections.

To predict the cell developmental trajectory from CSCs to HCC and identify genes associated with evolution and development, we performed pseudotime analysis. The pseudotime trajectory plot generated using Monocle3 (Figure 5B) revealed that CSCs served as the starting point of development for malignant hepatocytes. Along the pseudotime trajectory, malignant hepatocytes diversified into 12 distinct developmental subtypes, aligning with the results obtained from inferCNV and emphasizing the pronounced heterogeneity of malignant hepatocytes. Additionally, we conducted Monocle2 pseudotime analysis, and the findings are depicted in Figure 5C-D. Through the utilization of a spatial differential gene algorithm, we calculated the Moran's I spatial autocorrelation for genes and ranked them based on Moran's I. Subsequently, we performed fGSEA enrichment analysis for genes associated with development (Moran's I > 0.5). The results (Figure 5E) suggested the involvement of signaling pathways such as MAPK, PPAR, and leukocyte transendothelial migration in the regulation of CSCs development, particularly through pathways previously reported to be associated with p38 MAPK/HSPB1. Furthermore, we presented the expression profiles of selected genes highly correlated with development (Moran's I > 0.8) along the pseudotime trajectory (Figure 5F). Among these genes, ADH4, ATP5F1E, GAGE12H, and IGLV2-14 exhibited a tendency for high expression during the mid-stage of development. RACK1 and AGXT exclusively displayed high expression in the late stage of development. Metabolism-related genes such as AHSG, ALB, and APOE gradually increased in expression during the mid-late stage of development, indicating their stronger association with abnormal tumor metabolism. VCX3A and HSPB1 showed elevated expression only in the early stage of development, implying their close correlation with CSCs development. The UMAP dimensionality reduction density plot illustrating the mentioned genes is presented in Figure 5G.

### Weighted Gene Co-expression Network Analysis (WGCNA)

**Figure 6:**
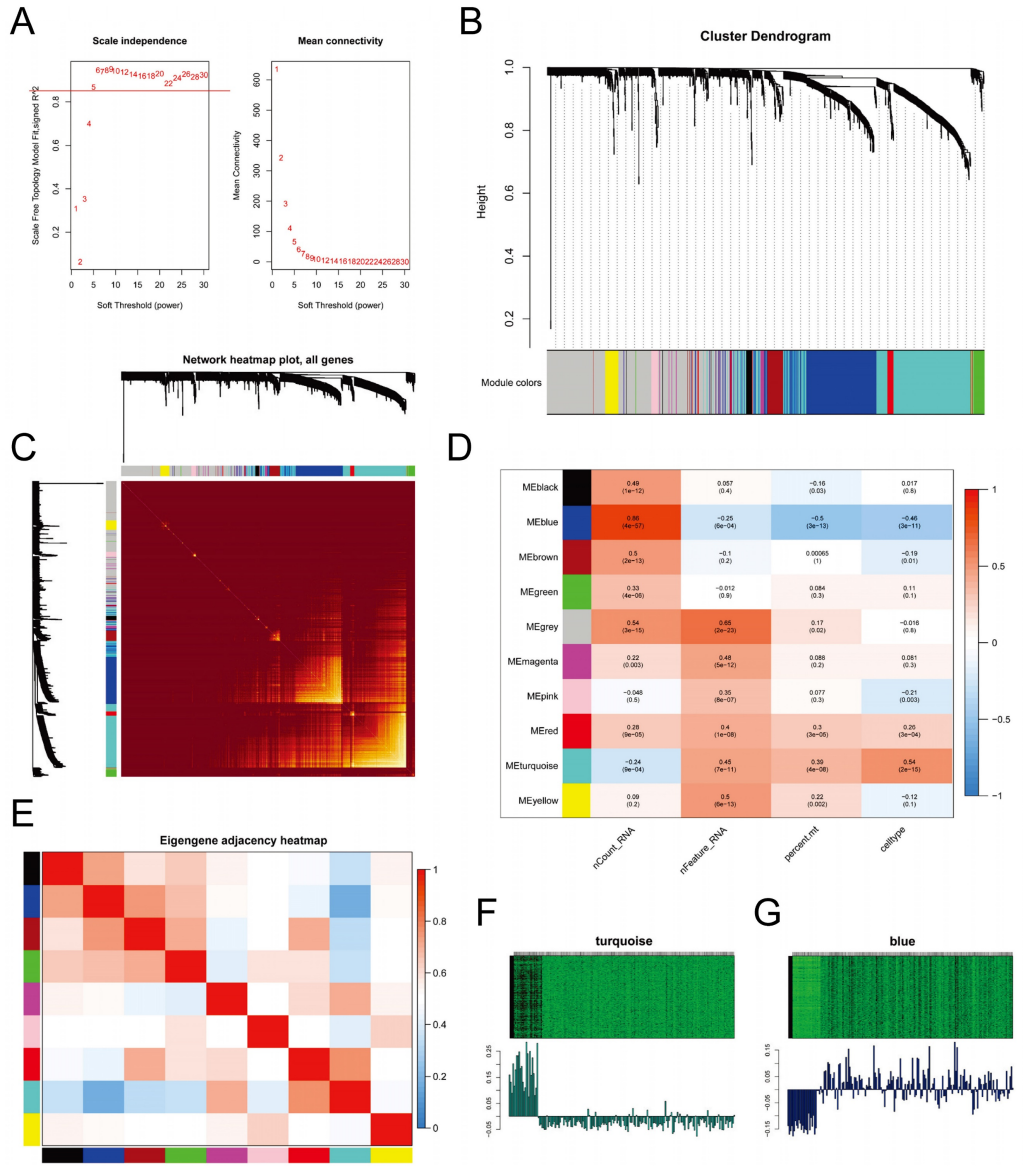
** Weighted Gene Co-expression Network Analysis.** A. Determination of the power of the network for connectivity based on the scale-free topology fit index (Left) and mean connectivity (Right); B. Cluster dendrogram displaying different gene modules in the weighted gene co-expression network; C. Heatmap of the topological overlap matrix (TOM) for all genes, with red indicating low overlap and yellow indicating high overlap; D. Heatmap showing the correlation between different gene modules and single-cell features; E. Heatmap displaying the co-expression relationships between different gene modules; F-G. Heatmaps illustrating the expression levels of feature genes in the MEturquoise module (F) and the MEblue module (G).

We employed WGCNA to identify gene expression modules. The optimal power value of 5 was determined based on the scale-free topology fit index (>0.85) and mean connectivity (Figure 6A). The clustering dendrogram and TOM heatmap of WGCNA are presented in Figure 6B-C. Additionally, we generated heatmaps illustrating the correlation between modules and phenotypes (Figure 6D) and between module eigengenes (MEs) and the expression levels of all genes (Figure 6E). The analysis revealed that the MEturquoise module (R = 0.54, *p*-value = 2e-15) and the MEblue module (R = -0.46, *p*-value = 3e-11) exhibited the strongest correlation with CSCs. Notably, these two modules displayed a negative correlation with each other. Further examination of the heatmap depicting characteristic gene expression levels (Figure 6F-G) uncovered that the MEturquoise module had a high expression in CSCs, whereas the MEblue module exhibited low expression.

### Construction and Validation of Clinical Prediction Model

**Figure 7:**
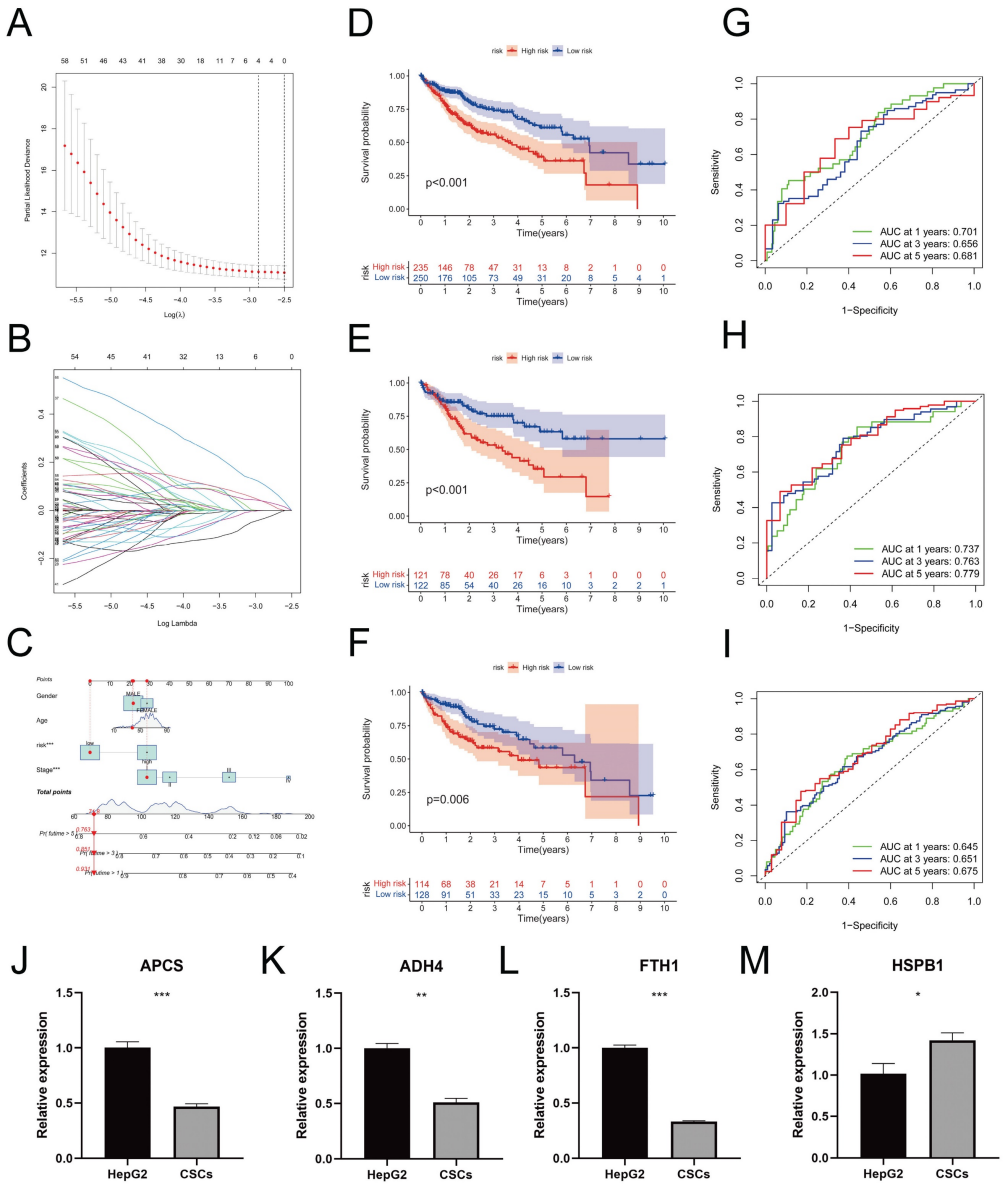
** Construction and Validation of Prognostic Model.** A. Distribution curve of coefficients in the LASSO regression model; B. 10-fold cross-validation for variable selection in the LASSO regression model; C. Nomograms validated by calibration curves; D-F. Kaplan-Meier survival curves for different risk score groups in all samples (D), the training set (E), and the test set (F); G-I. Receiver Operating Characteristic (ROC) curves for 1-year, 3-year, and 5-year survival in all samples (G), the training set (H), and the test set (I). J-M. In comparison to CSCs, the relative mRNA expression levels of APCS (J), ADH4 (K), FTH1 (L), and HSPB1 (M) in HepG2 cells. (* *P* < 0.05, ** *P* < 0.01, *** *P* < 0.001).

Using LASSO regression analysis (Figure 7A) and conducting 10-fold cross-validation (Figure 7B), we identified four genes associated with prognosis and established a risk prediction model as follows: Risk Score = Expression_APCS_ × (-0.1036) + Expression_FTH1_ × (-0.2915) + Expression_HSPB1_ × (0.1010) + Expression_ADH4_ × (-0.0755). Subsequently, we incorporated the clinical features of the samples, including age, gender, and tumor stage. Furthermore, calibrated nomograms were obtained (Figure 7C) through calibration curve validation. The nomograms exhibited predictive accuracies of 93.1%, 85.1%, and 76.3% for 1-year, 3-year, and 5-year overall survival (OS) predictions, respectively. The calibration curves demonstrated a strong agreement between the predicted and actual OS. Risk scores were calculated for each sample, and all samples, as well as the training and testing cohorts, were divided into Risk-High and Risk-Low groups based on the median risk score. Kaplan-Meier survival curves for OS in the different cohorts are presented in Figure 7D-F, indicating significantly lower OS in the Risk-High group compared to the Risk-Low group. Consistent results were observed between the training and testing cohorts. The ROC curves (Figure 7G-I) indicated that the model exhibited high predictive value. Heatmaps illustrating the expression levels of the four prognostic-related genes in all samples, the training cohort, and the testing cohort can be found in Figure S4A-C. Additionally, the distribution of risk scores (Figure S4D-F) and survival status (Figure S4G-I) in different cohorts were presented. The RT-qPCR results (Figure 7J-M) reveal a significant increase in the expression levels of APCS (*P* = 0.0009), ADH4 (*P* = 0.0011), and FTH1 (*P* < 0.0001) in HepG2 cells when compared to CSCs. Conversely, the expression level of HSPB1 (*P* = 0.0284) is decreased. These results align with the prognostic model.

## Discussion

Currently, the standard treatment for HCC has shifted towards a combination of immune therapy and targeted therapy. The IMbrave150 study has shown that the combination of atezolizumab and bevacizumab is now a first-line treatment option for unresectable, locally advanced, or metastatic hepatocellular carcinoma (uHCC)^35^. According to the latest ASCO 2023 research report^36^, combining a TIGIT inhibitor with atezolizumab and bevacizumab nearly doubled the median progression-free survival (11.1 months vs. 4.2 months), providing a promising new first-line treatment option for uHCC. Despite advancements in systemic treatment strategies for uHCC, the survival outcomes for patients with uHCC remain unfavorable.

During systemic treatment, HCC is prone to developing drug resistance and metastasis, with CSCs playing a prominent role. CSCs contribute to the high heterogeneity of tumors, drug resistance, and recurrence due to their robust self-renewal capacity. Moreover, CSCs regulate various cells within the tumor microenvironment (TME), thereby altering cellular metabolism patterns and intercellular communication. These changes lead to immune evasion, T-cell exhaustion, and the establishment of a hypoxic TME. Our research findings highlight that CSCs not only modulate other cells, particularly macrophage subtypes, through specific ligand-receptor interactions to reshape the TME, but they also possess distinctive metabolic profiles that differ significantly from both tumor and normal cells. This suggests that metabolism plays a pivotal role in CSCs-mediated regulation of the TME. Furthermore, CSCs can promote the progression of HCC by activating specific signaling pathways.

The influence of CSCs on the TME encompasses various factors, including physicochemical and metabolic characteristics, stromal cells, and immune cells^37^. Our research findings demonstrate that CSCs display heightened activity in hypoxic pathways, as hypoxia plays a critical role in maintaining the stem-like properties of tumor cells. Hypoxia contributes to the enhancement of stemness in HCC through HIF1α- and HIF2α-dependent mechanisms, while also supporting the persistence of CD24+ CSCs^38^. Additionally, hypoxia activates HIF1α via the IL-6/STAT3 signaling pathway, resulting in increased CD133 expression and the maintenance of tumor cell stemness^39^. Metabolic analysis further reveals that both CSCs and non-CSC HCC cells exhibit elevated activity in glycolysis/gluconeogenesis pathways. However, CSCs exhibit overall low glucose metabolism with noticeable glucose deprivation. Glucose deprivation is a significant physicochemical factor in maintaining the stemness of tumor cells. Studies suggest that glucose deprivation induces abnormal fucosylation of membrane glycoproteins mediated by FUT1, thereby sustaining tumor cell stemness through the AKT/mTOR/4E-BP1 signaling pathway^40^. Notably, CSCs and non-CSCs HCC demonstrate notable disparities in fatty acid metabolism, the PPAR signaling pathway, and the peroxisome proliferator-activated receptor pathway, indicating potential involvement of CSCs in lipid metabolism within the tumor tissue. Existing evidence suggests that the overexpression of OCTN2 enhances PGC-1α-mediated fatty acid oxidation and oxidative phosphorylation, thereby promoting the stemness of HCC^41^. Furthermore, abnormal lipid metabolism can also contribute to the maintenance of HCC stemness through inflammation and the activation of oncogenes^42^, ^43^.

Cell communication results indicate that CSCs primarily regulate the TME by secreting MIF (MIF-CD74/CXCR4, MIF-CD74/CD44) 44, 45 or MDK (MDK-SDC2, MDK-NCL), which acts on stromal cells and immune cells, particularly myeloid cells and CAFs, thereby modulating the TME. CSCs can enhance the function of myeloid-derived suppressor cells (MDSCs) through the MIF/CXCR2 pathway, resulting in the mediation of an immunosuppressive microenvironment^46^. Additionally, the MIF/CXCR4 pathway is considered crucial for the recruitment of mesenchymal stem cells^47^. Single-cell transcriptomic studies have demonstrated that tumor cells can also affect CAFs^48^ and endothelial cells^49^ through the MDK-NCL pathway, contributing to an immunosuppressive microenvironment. Signaling pathways associated with stemness play a significant role in systemic treatment resistance^50^. Our findings suggest the presence of aberrantly activated Hedgehog and MAPK signaling pathways in CSCs. Studies have confirmed the involvement of the Hedgehog signaling pathway^51^, ^52^ and MAPK signaling pathway^53^, ^54^ in regulating CSC differentiation and drug resistance. Importantly, by combining Monocle3 pseudotime analysis and prognostic modeling, we have discovered that HSPB1 acts as a regulatory factor in CSCs development, and its high expression indicates a poor prognosis in HCC. Therefore, HSPB1 as downstream targets of the p38 MAPK signaling pathway and upstream activators of the NF-κB signaling pathway^55^, p38 MAPK/HSPB1/NF-κB is likely a key pathway involved in CSCs development, preliminary studies have provided initial support for this hypothesis^56^-^58^, and further validation is warranted.

We focused on analyzing the interactions between CSCs and the HCC microenvironment. Although we did not conduct in-depth research on stromal cells and immune cells within the HCC microenvironment, we observed intriguing phenomena that warrant further investigation. These phenomena include the interactions between macrophages and CAFs, alterations in gene expression profiles and metabolism in exhausted T cells, and the role of B cells in the tumor microenvironment (TME). Recent studies have substantiated the significance of these phenomena in terms of research value^59^-^63^. Nevertheless, it is important to acknowledge the limitations of this study. Building upon the interesting findings, our future research will employ single-cell transcriptomics and spatial transcriptomics methodologies to delve deeper into these areas of inquiry.

## Conclusions

In this study, we utilized scRNA-Seq data from HCC tissues to elucidate the mechanisms underlying the interaction between CSCs and the TME through comprehensive omics analyses. These analyses encompassed cell communication, cell trajectories, and the activation of aberrant signaling pathways that provides a holistic understanding of the pivotal role played by CSCs in the TME. Moreover, by integrating bulk RNA-Seq data, we developed a clinical prognostic model. Additionally, our research shed light on intriguing phenomena involving stromal cells and immune cells within the TME, thereby necessitating further in-depth investigations.

## Supplementary Material

Supplementary figures and tables.
