# Supplementary material for: Single-cell RNA-Seq Elucidates the Crosstalk Between Cancer Stem Cells and the Tumor Microenvironment in Hepatocellular Carcinoma
Source: J Cancer. 2024 Jan 1;15(4):1093–109. doi: 10.7150/jca.92185 (PMC10788724; doi:10.7150/jca.92185)
Supplement: Supplementary file 1 — Supplementary figures and tables. [file jcav15p1093s1.pdf]

Figure S1: Preprocessing of scRNA-Seq Data

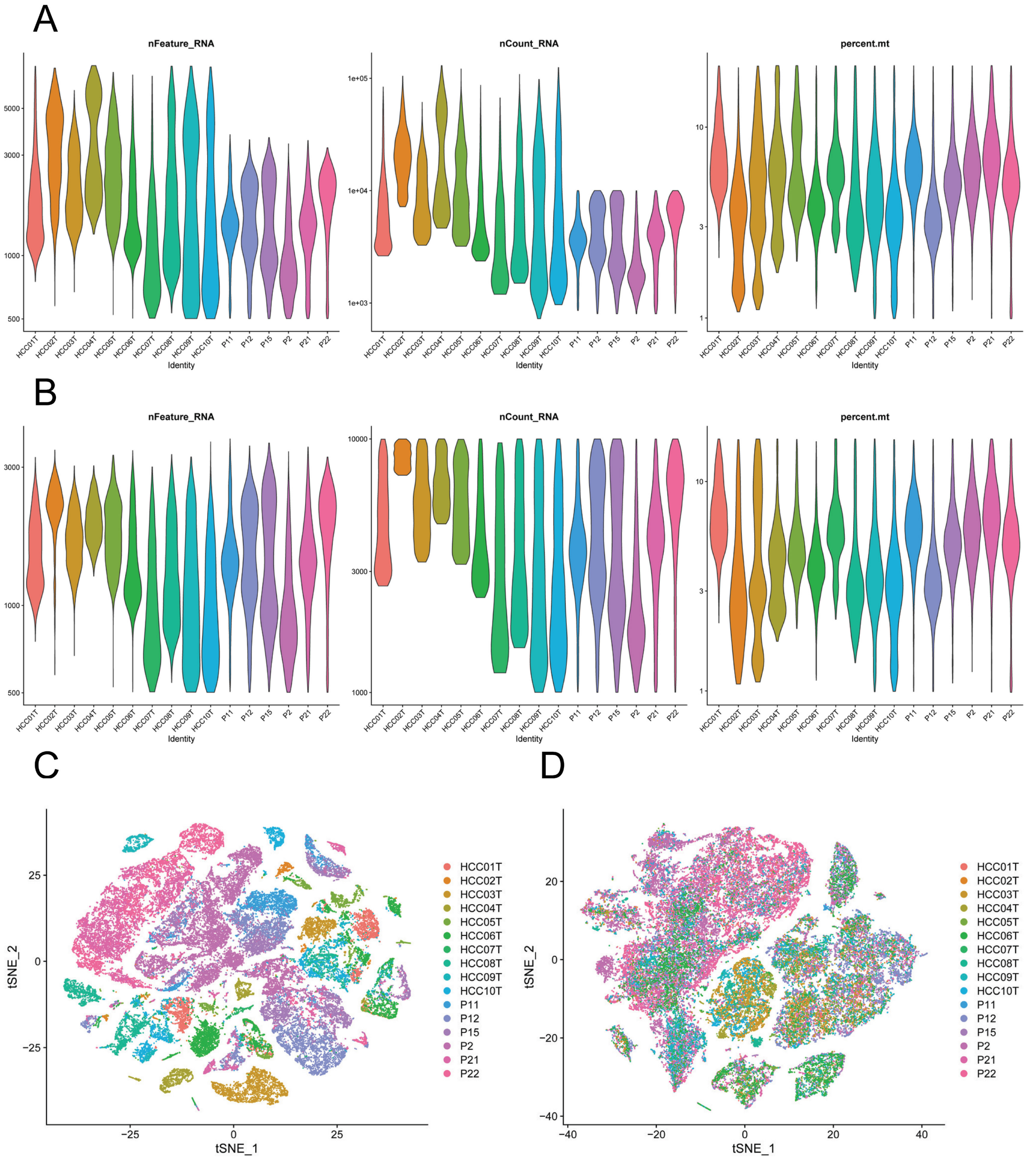

A. Features of the pre-quality control data in each cell, including the count of RNA molecules (nFeature), total gene expression count per cell (nCount), and proportion of mitochondrial genes (percent.mt); B. Features of the post-quality control data, including nFeature, nCount, and percent.mt; C. t-SNE dimensionality reduction clustering plot prior to normalization; D. t-SNE dimensionality reduction clustering plot after batch effect correction.

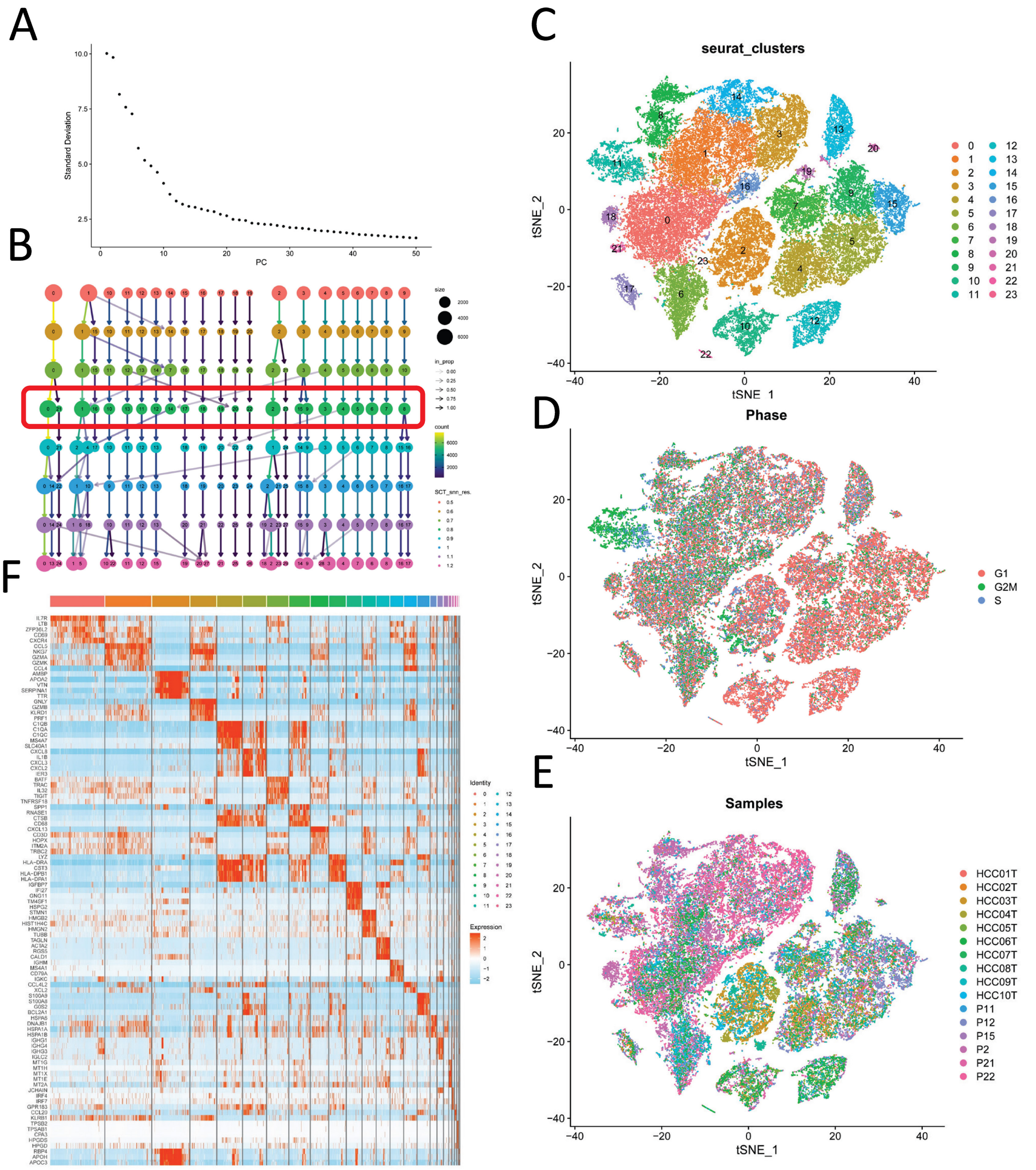

Figure S2: Clustering and Differential Gene Expression Analysis of Dimensionality-Reduced Clusters

A. Scree plot of principal component analysis (PCA); B. Determination of the appropriate resolution for clustering (0.5-1.2) based on the number of clusters; C-E. t-SNE dimensionality reduction clustering plots depicting distinct clusters, cell cycle, and sample features; F. Heatmap showing the top 5 differentially expressed genes (DEGs) in each cluster.

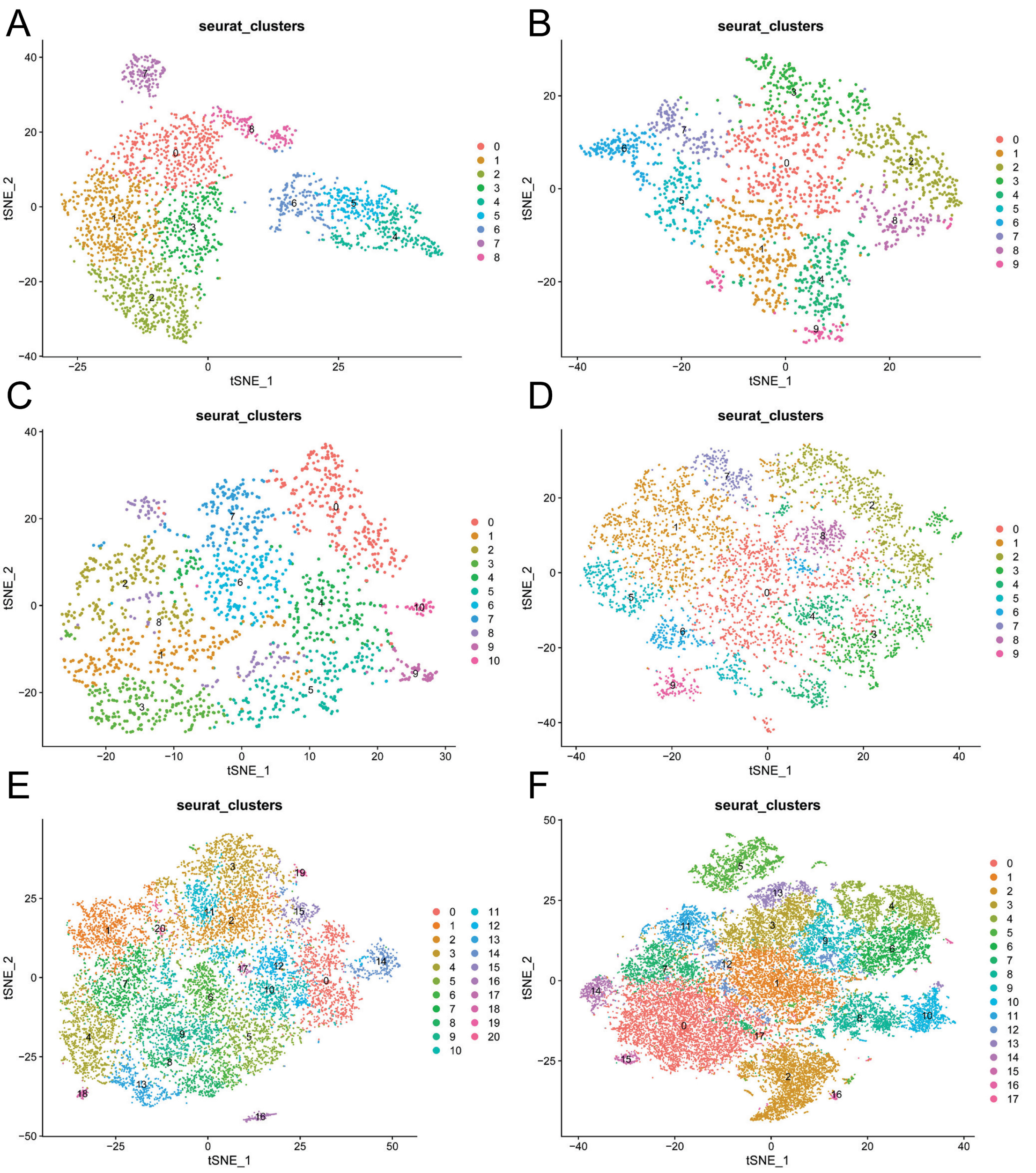

Figure S3: Secondary t-SNE Dimensionality Reduction Clustering Results of Mian Cell Types

A-F. t-SNE dimensionality reduction clustering plots illustrating cluster features of B cells, endothelial cells, fibroblasts, hepatocytes, myeloid cells, and NK/T cells, respectively.

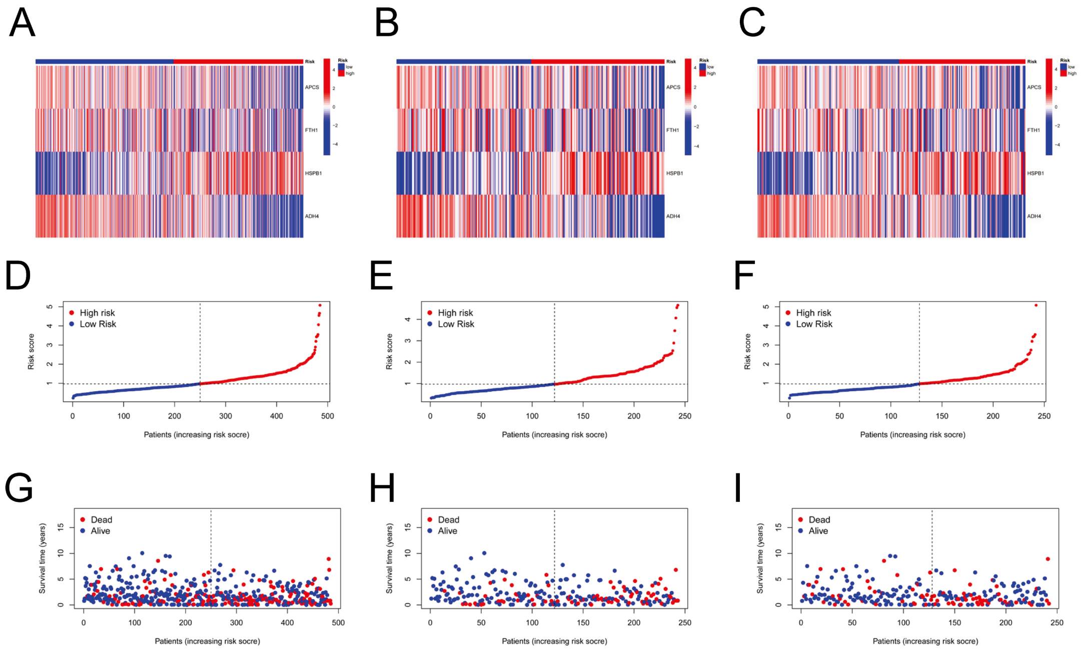

Figure S4: Sample Features in the Prognostic Model

A-C. Heatmaps presenting the expression levels of prognostic-related genes in all samples (A), the training set (B), and the test set (C); D-F. Distribution of risk scores of samples (D), the training set (E), and the test set (F); G-I. Survival status of all samples (G), the training set (H), and the test set (I).

TableS1 Clinical Characteristics of Enrolled HCC Patients.

Patient ID	Gender	Age	Histopatho logy	Primary/ Metastasis	Infect ion	TNM Staging			
						T	N	M	TNM
HCC01	Male	66	HCC	Primary	HBV	T1	N0	M0	I
HCC02	Male	65	HCC	Primary	HBV	T1	N0	M0	I
HCC03	Male	66	HCC	Primary	HCV	T1	N0	M0	I
HCC04	Male	60	HCC	Primary	HCV	T2	N0	M0	II
HCC05	Male	65	HCC	Primary	None	T3	N0	M0	IIIA
HCC06	Female	64	HCC	Primary	HBV	T3	N0	M0	IIIA
HCC07	Male	48	HCC	Primary	None	T4	N0	M0	IIIB
HCC08	Male	64	HCC	Primary	None	T4	N0	M0	IIIB
HCC09	Male	48	HCC	Primary	HBV	T4	N0	M0	IV
HCC10	Male	53	HCC	Primary	HBV	T4	N0	M1	IV
P2	Male	56	HCC	Primary	None	/	/	M1	IV
P11	Male	52	HCC	Primary	None	/	/	M1	IV
P12	Male	64	HCC	Primary	HBV	/	/	M1	IV
P15	Male	58	HCC	Primary	HBV	/	/	M1	IV
P21	Female	63	HCC	Primary	HBV	/	/	M1	IV
p22	Female	49	HCC	Primary	None	/	/	M1	IV

TableS2 Primer Sequences

	Sequencing
APCS	Forward: 5’- TAGTGATCTCTCTCGTGCCTAC-3’<br>Reverse: 5’- GCCGGGAACTTTTCGATAACTT-3’
ADH4	Forward: 5’- AGTTCGCATTCAGATCATTGCT-3’<br>Reverse: 5’- CTGGCCCAATACTTTCCACAA-3’
FTH1	Forward: 5’- CCCCCATTTGTGTGACTTCAT-3’<br>Reverse: 5’- GCCCGAGGCTTAGCTTTCATT -3’
HSPB1	Forward: 5’- ACGGTCAAGACCAAGGATGG-3’<br>Reverse: 5’- AGCGTGTATTTCCGCGTGA-3’

β-Actin	Forward: 5’-AGGATGCAGAAGGAGATCAC-3’<br>Reverse: 5’-TGTAACGCAACTAAGTCATAG-3’

TableS3 Reverse Transcription Reaction Mixture

Reagents	Quantities/μL
5×gDNA Eraser Buffer	2
gDNA Eraser	1
Total RNA	4
RNase Free dH2O	3
Total	10

TableS4 Reverse Transcription Reaction System

Reagents	Quantities/μL
Reverse transcription reaction mixture	10
PrimeScript RT Enzyme Mix I	1
RT Primer Mix	4
5×PrimeScript Buffer 2	4
RNase Free dH2O	1
Total	20

TableS5 RT-qPCR Reaction System

Reagents	Quantities/μL
SYBR Premix Ex Taq™II	5
Forward Primer(10μM)	0.2
Reference Primer(10μM)	0.2
Template DNA	1
ddH2O	3.6
Total	10

TableS6 Formulation for Serum-free Culture Medium

Reagents	Quantities
DMEM/F12	19 mL
B27	0.4 mL

Nonessential amino acids	0.2 mL
Sodium pyruvate	0.2 mL
EGF	40 μL
bFGF	40 μL
penicillin	4 μL
streptomycin	4 μL
Total	20 mL
